# Validating the Turkish version of the Weinstein noise sensitivity scale:effects of age, sex, and education level

**DOI:** 10.3906/sag-1908-44

**Published:** 2020-06-23

**Authors:** Melis KESKİN YILDIZ, Yusuf Kemal KEMALOĞLU, Yetkin TUAÇ, Güven MENGÜ, Recep KARAMERT, Çağıl GÖKDOĞAN

**Affiliations:** 1 Department of Audiology, Faculty of Health Sciences, Gazi University, Ankara Turkey; 2 Deaprtment of Otolaryngology & Audiology Subdivision, Faculty of Medicine, Gazi University, Ankara Turkey; 3 Department of Statistics, Faculty of Science, Ankara University, Ankara Turkey; 4 Department of Western Languages and Literatures, Faculty of Letters, Ankara Hacı Bayram Veli University, Ankara Turkey; 5 Department of Otolaryngology, Faculty of Medicine, Gazi University, Ankara Turkey; 6 Department of Otorhinolaryngology, Faculty of Medicine, Muğla Sıtkı Koçman University, Muğla Turkey

**Keywords:** Noise annoyance, noise sensitivity, questionnaire survey, Weinstein’s noise sensitivity scale

## Abstract

**Background/aim:**

Subjective noise sensitivity (SNS) is not only one of the most important predictors of perceived noise annoyance for the subjects exposed to the noise, but also strongly related with some psychological conditions and poor health perception of the subjects. Weinstein’s Noise Sensitivity Scale (WNSS) is widely used for measuring of SNS.

**Materials and methods:**

In this study we adapted WNSS to Turkish (Tr-WNSS) and evaluated its psychometric properties and effect of age, sex and education level on SNS.

**Results:**

The study included 105 males and 105 female subjects between 18 and 55 years of age.

**Conclusion:**

Our data demonstrated that Tr-WNSS was a reliable, valid, and invariant scale for the Turkish population, and not affected by sex, age and education level.

## 1. Introduction

Noise has been described as unwanted sound. The American Hearing and Speech Association (ASHA) points out that one person’s music is another person’s noise [1]. However, a part of population appears to be more prone to perceive sound as a negative condition and present strong emotional reaction to noisein the same acoustical conditions [2–7]. It has been reported that the subjects with negative tendency to sound are resistant to adaptation to noise for longer periods of time [3–5,7], and that it is related with a genetic or familial tendency [8]. This concept has been named as subjective noise sensitivity (SNS) [5,6] or noise annoyance susceptibility [2].

The researchers clearly presented that SNS was a predictor of noise annoyance [5,7,9]. It is known that some clinical conditions such as head trauma, migraine headaches, facial paralysis, otologic infections and surgeries such as stapedectomy, and some medicines are also related with increasing annoyance from aloud sound (hyperacusis) [10,11]. Although the association of SNS with hyperacusis or phonophobia is still an unanswered question, the SNS concept summarized above appears to be a consistent and stabile personality traitin the subjects without such a clinical problem. No relationship of SNS with auditory acuity was reported [2,5,12,13]. It was also documented that noise sensitive people did not have more experience of exposition to noise [14].

However, it has been reported that SNS is related with behavioral differences, tendencies towards some psychological conditions, poor health perception, and social differences. Weinstein[3]reported that theywere lower in scholastic ability, felt less secure in social interactions, and had a greater desire for privacy. Weinstein [4] and Stansfeld [5,12] pointed out that people with SNS were more sensitive to sensory stimuli around them and hence more critical of their environmental quality. Stansfeld [5,15], Öhrström et al. [16], Belojevic and Jakovlevic [6], and Stansfeld and Shipley [17] pointed out a tendency toward neuroticism, depressive symptoms, and psychological distress. Furthermore, Babisch et al. [18], Fyhri and Klaeboe [19], and van Kamp and Davis [11] reported higher cardiovascular problems, while Stansfeld and Shipley [17] documented only a meaningful association between SNS and angina in people from lower employment grades. Besides, Shepherd et al. [20] found that SNS was negatively associated with self-reported health-related quality of life scores. Stansfeld and Stanley[17] recently documented that SNS has been identified as a predictor of mental ill-health, while Schreckenberg et al. [21] reported an association of SNS with physical health. These data point out that SNS is an important predictor not only for noise annoyance but also for health perception of the subjects and their predisposition to the psychological problems. 

Although the studies presented that environmental high noise appears to be important problem in Turkey [22–24] and there are some noise-annoyance questionnaires adapted to Turkish [23,25] and developed in Turkish [24], noise sensitivity has never been subject to any research in the Turkish population as much as we found in the English and Turkish literature. For the measurement of SNS, long and short questionnaires have been proposed [7]; the Weinstein noise sensitivity scale (WNSS) has been one of the most widely used standard questionnaires with high validity, reliability, and internal consistency [3,4,26,27]. In this study we aimed to adapt WNSS to Turkish and looked for its reliability, internal consistency, factor structure, and furthermore its stability regarding sex, age, and educational level in the Turkish population without any otologic and neuro-otologic problem.

## 2. Material and method

This study was approved by the clinical research ethics board in Gazi University (Approval date and no: 23.03.2015, 176). This research was conducted at the audiology department of the university hospital.

In the first step of the study, WNSS [3] was translated into Turkish by 2 of the authors and translated back to English. Then, the preliminary Turkish version was evaluated in a pilot study including 20 interviewers, and it was noticed that only small changes were needed, because all items were found to be clear in Turkish and understanding of the interviewers was in accordance with the original target of each question. After these small corrections, the Turkish version of WNSS (Tr-WNSS), which is comprised 21 items with a 6-point scale ranging from 1 (agree strongly) to 6 (disagree strongly) was completed. Hence, summing up the scores of all items created a noise sensitivity score (NSS) of each subject. For test reliability, Tr-WNSS questionnaire was refilled out by 64 subjects after a 15-day interval. The test/retest reliability was assessed with Pearson’s test. Then, validation of the scale was calculated with confirmatory factor analysis (CFA). The internal consistency for Tr-WNSS was calculated with Cronbach’s alpha (α) coefficient. In order to test homogeneity of variances, Levene’s test was used. According to result of Levene’s tests, homogeny or nonhomogeny group of variances decided and independent 2 sample t-test was conducted. By using analysis of variance test, the differences between groups were assessed.

In the second step of the study, Tr-WNSS was applied to 210 subjects (M: 105, F: 105) aged between 18 and 55 years. Inclusion criteria were as follows: no chronic health problem causing functional impairment in daily life (only exception was use of eyeglasses), no craniofacial abnormality or congenital disorders, and normal hearing as personal perception without history of any otologic and/or neuro-otologic disorders. 

NSS was calculated for each subject and compared between i) males and females, ii) age groups (age subgroup-1: the subjects younger than 35 years of age; age subgroup-2: the subjects between 35 and 55 years of age), and iii) education groups (education subgroup-1: elementary, secondary and high school; education subgroup-2: 2-years of higher education after high school or Bachelor’s degree). Furthermore, correlation of NSS with age was tested.

Then according to NSS, the lowest (lowest 30%) and highest (highest 30%) SNS subgroups were found, and the lower and upper SNS groups were compared regarding sex, age, and education level in addition to NSS. 

## 3. Results

In whole study group, mean age of 105 male subjects (36.57 years, minimum: 19; maximum: 56) was not different from those of females (35.19 years, minimum: 19; maximum: 62). Of 210 subjects, 111(M: 53, F: 58) were younger than 35 years of age, and 149 (70.95%) were graduates of 2-years of higher education after high schoolor Bachelor’s degree or higher (Table 1).

**Table 1:** Sex and age data of the study group and subgroups.

	Males	Females	Total
Total	n: 105 36.57 ± 10.94*	n: 105 35.19 ± 9.4*	n: 210 35.88 ± 10.2*
- Age subgroups			
35>	n: 53 27.26 ± 3.95*	n: 58 28.22 ± 3.62*	n: 111 27.77 ± 3.8*
35≤	n: 52 46.06 ± 6.82*	n: 47 43.79 ± 6.86*	n: 99 44.98 ± 6.9*
- Education subgroups			
Elementary to high school	n: 22 37.5 ± 10.92*	n: 39 33.54 ± 7.51*	n: 61 34.97 ± 9.12*
Two-years or bachelor’s degree or higher	n: 77 36.51 ± 11.01*	n: 65 36.28 ± 10.33*	n:142 36.4 ± 10.67*

* mean age, years.

At the first step of the study, by using the data of 64 subjects (37 males with mean age of 33.95 ± 3.37 months and 27 females with mean age of 34.23 ± 9.88), it was found that test reliability was high by Tr-WNSS (Pearson’s test, r: 0.92, P < 0.001). Levine test presented that the data were distributed in accordance with normal distribution.

The factor structure of the 21-item scale was tested by using CFA. The CFA was conducted using IBM SPSS AMOS 26 software. We follow the same strategy for the factor model case with Senese et al. [28]. Items 2, 4, 5, 6, 7, 10, 11, 13, 16, 17, 18, 19, and 21 were specified to the first factor, while items 1, 3, 8, 12, 14, 15, and 20 were specified to the second factor. From this setting of the items, we summarized the following results in Table 2. 

**Table 2:** Factor loadings of the items.

Item	Factor	Estimate
s2	Factor 1	.342
s4	Factor 1	.440
s5	Factor 1	.460
s6	Factor 1	.541
s7	Factor 1	.580
s10	Factor 1	.550
s11	Factor 1	.158
s13	Factor 1	.565
s16	Factor 1	.564
s17	Factor 1	.512
s18	Factor 1	.552
s19	Factor 1	.674
s9	Factor 1	.502
s21	Factor 1	.758
s1	Factor 2	.357
s3	Factor 2	.406
s8	Factor 2	.597
s12	Factor 2	.316
s14	Factor 2	.640
s15	Factor 2	.400
s20	Factor 2	.476

According to Table 3 the default model fit has the following parameters. χ² = 267.621 with df = 169 which means that the default model is statistically significant. We can also support the result from CMIN/DF ratio is below 3, which is a good metric for an acceptable model (CMIN/DF = 1.584). We do note that the RMSEA is below the accepted threshold (<0.10) for the default model (RMSEA = 0.054). The CFI value is 0.888 which is between traditional bounds and it is acceptable for the default model. The GFI value is a little below than the acceptable value but it is in satisfactory limits (GFI = 0.892). The AGFI value is above the accepted threshold (>0.80), which is 0.866. The SRMR value is way below than .09 for our model (SRMR = 0.00). The TLI parameter value is close to 1 (TLI = 0.874), which indicates a very good fit for the default model. Also, according to the AIC values, the default model has the minimum value among the others (AIC = 349.621). This means that the default model has the best fit. All other values for the parameters of the CFA results can be seen in the Table 3. Figure is the diagram of the CFA analysis which shows the relations and the correlations coefficients between the factors. Our results support the conclusion that the 2 latent factors are strong reflections of the associated observed variables.

**Table 3:** The summary of the CFA model results

CMIN					
Model	NPAR	CMIN	DF	P	CMIN/DF
Default model	41	267.621	169	0	1.584
Saturated model	210	0	0		
Independence model	20	1073.144	190	0	5.648
RMR, GFI					
Model	RMR	GFI	AGFI	PGFI	
Default model	0.164	0.892	0.866	0.718	
Saturated model	0	1			
Independence model	0.632	0.467	0.41	0.422	
Baseline comparisons					
Model	NFI	RFI	IFI	TLI	CFI
	Delta1	rho1	Delta2	rho2	
Default model	0.751	0.72	0.891	0.874	0.888
Saturated model	1		1		1
Independence model	0	0	0	0	0
Parsimony-adjusted measures					
Model	PRATIO	PNFI	PCFI		
Default model	0.889	0.668	0.79		
Saturated model	0	0	0		
Independence model	1	0	0		
NCP					
Model	NCP	LO 90	HI 90		
Default model	98.621	58.024	147.148		
Saturated model	0	0	0		
Independence model	883.144	783.968	989.814		
FMIN					
Model	FMIN	F0	LO 90	HI 90	
Default model	1.28	0.472	0.278	0.704	
Saturated model	0	0	0	0	
Independence model	5.135	4.226	3.751	4.736	
RMSEA					
Model	RMSEA	LO 90	HI 90	PCLOSE	
Default model	0.053	0.041	0.065	0.337	
Independence model	0.149	0.141	0.158	0	
AIC					
Model	AIC	BCC	BIC	CAIC	
Default model	349.621	358.78	486.852	527.852	
Saturated model	420	466.915	1122.893	1332.893	
Independence model	1113.144	1117.612	1180.086	1200.086	
ECVI					
Model	ECVI	LO 90	HI 90	MECVI	
Default model	1.673	1.479	1.905	1.717	
Saturated model	2.01	2.01	2.01	2.234	
Independence model	5.326	4.852	5.836	5.347	

When participants were asked 21 questions, the CFA was used for validity of Turkish scales. CFA results showed that 2-factor model fits the model according to the significance value P < 0.05 (Table 2). These 2 factors are highly correlated to each other (r = 0.656; P < 0.05). As supported by different researches, the scale can be considered substantially unidimensional because the second factor can be accounted for as an effect of the direction of item wording more than an expression of a different dimension. This was also confirmed by the strong correlation between the 2 factors. 

At the second step of the study, it was observed that the mean NSS value was 88.72 ± 17.97 for 210 volunteers, and 89.22 ± 16.21 (minimum: 48 and maximum: 124) for males and 88.23 ± 19.63 (minimum: 30 and maximum: 119) for females (Table 4). No statistical difference between males and females and between age subgroups and between education subgroups was detected (Student-t test, P > 0.05) (Table 4). Pearson’s correlation analysis declared that NSS was not correlated with age in either total group or male and female subgroups (Pearson’s test, P > 0.05).

**Table 4:** Noise sensitivity scores of the study group and subgroups.

	Males	Females	Total
Total	n: 10589.22 ± 16.21	n: 10588.23 ± 19.63	n: 21088.72 ± 17.97
- Age subgroups			
35>	n: 5388.23 ± 16.31	n: 5890.09 ± 18.85	n: 11188.19 ± 18.42
35≤	n: 5290.23 ± 16.21	n: 4785.94 ± 20.53	n: 9989.2 ± 17.63
- Education subgroups			
Elementary to high school	n: 2292.14 ± 11.58	n: 3988.03 ± 19.73	n: 6189.51 ± 17.24
Two-years or bachelor’s degree or higher	n: 7787.77 ± 17.19	n: 6588.32 ± 19.88	n:14288.02 ± 18.4

It was observed that there were 60 subjects (M: 32, mean age: 35.81 ± 9.82, min: 19, max: 54, F: 28, mean age: 35.93 ± 10.97, min: 21, max: 55) in the lowest SNS group (NSS was 81 or lower) and 60 subjects (M: 29, mean age: 35.86 ± 11.09, min: 23, max: 55, F: 31, mean age: 34.23 ± 9.01, min: 19, max: 55) in upper SNS group (NSS was 99 or higher). NSS was significantly higher in the upper SNS group (108.73 ± 6.66) than the lower SNS group (66.23 ± 11.8) (Student-t test, P < 0.001). A statistically significant difference between upper and lower SNS groups was also found in male (70.0 ± 9.31 vs. 107.9 ± 7.54, P < 0.001) and female (61.93 ± 12.99 vs. 109.52 ± 5.94) subgroups. No statistical difference in age was detected between the upper and lower groups (Student-t test, P > 0.05). No difference in male/female rate and education and age subgroups was detected between the upper and lower SNS groups (*x*2 test, P > 0.05) (Table 5). Besides, it was observed that 30.48% of all male subjects of the study were in the upper SNS group while it was 26.67% for females (*x*2 test, P > 0.05). Of 111 subjects who were younger than 35 years of age, 27.93% was in the upper group; 29.29% of 99 subjects who were 35 years or older were in the upper SNS group. It was seen that 24.6% of 61 subjects in the lower education group were in the upper SNS group; this rate was 31.69% of 142 subjects in higher education groups (*x*2 test, P > 0.05).

**Table 5:** The subjects in upper and lower subjective noise sensitivity (SNS) groups.

	Upper SNS			Lower SNS		
	Males	Females	Total	Males	Females	Total
Total	32 (53.33%)	28 (46.67%)	60 (100%)	29 (48.33%)	31 (51.66%)	60 (100%)
- Age subgroups						
35>	17 (54.84%)	14 (45.16%)	31 (100%)	15 (42.86%)	20 (57.14%)	35 (100%)
35≤	15 (51.72%)	14 (48.28%)	29 (100%)	14 (56%)	11 (44%)	25 (100%)
- Education subgroups						
Elementary to high school	5 (33.33%)	10 (66.67%)	15 (100%)	5 (33.33%)	10 (66.67%)	15 (100%)
Two-years- or bachelor’s degree or higher	27 (60%)	18 (40%)	45 (100%)	20 (44.44%)	25 (55.56%)	45 (100%)

## 4. Discussion

Garrioch [29] reported that the prosperous Greek colony Sybaris in Italy prohibited industrial noises in residential areas in 720 BCE. The historical papers present that not only noise exposure but also noise annoyance increases in modern life since the time of Sybaris [29–32]. Belojevic et al. [7] say that noise affects the person who is “doing nothing” by annoying him, while it affects the person who works by impairing his performance or making it more costly in terms of increased effort. Therefore, during the last century, not only annoyance but also its impact on human performance and interpersonal behavior has been subjected to studies by social psychologists [33]. SNS has been documented as a predictor or modulator for noise annoyance level when exposed [2–7,9]. In this study, we adapted WNSS to Turkish (Tr-WNSS) to make it available for multidisciplinary researches related to noise. Our data documented that Tr-WNSS was a convenient tool with strong internal and external consistency in the Turkish population, as reported in other adaptation studies of WNSS to non-English languages including Swedish [26], German [34], Japanese [35], Persian [27], and Italian [28]. Hence, WNSS could be used in Turkish for predicting personal behavior in the case of noise exposure without language barrier. 

Besides, although it has been developed with college student samples by Weinstein [3], the studies documented that it is valid and convenient in other populations, such as the depressed subjects and hospital staff between 18 and 65 years of age [5], the adult residents living close to heavy traffic roads in Japan between 20 and 70 years of age [35], nonindustrial employees between 17 and 76 years of age in Iran [27], and adults living in the neighborhoods of southern Italy [28]. In our study, Tr-WNSS presented a convenient assessment of SNS in adults between 18 and 55 years of age so that the highest 30% could be significantly different from the lowest 30%.

Nevertheless, it could be said that the average score in our study revealed some differences from previous studies. While it was 54.6 ± 12.1 (with 1–6 rating) in the study of Weinstein [3], it was found to be 89.41 ± 17.38 (with 1–6 rating) in our study. The other studies conducted on university students in Switzerland and Germany reported 57.5 ± 12.61 (with 0–5 rating; it must have been 69 by 1–6 rating) and 63.08 ± 14.07, respectively [26,34]. Furthermore, Stansfeld [5] used WNSS (with 1–6 rating) in depressed subjects and control group composed of the hospital staff between 18 and 65 years of age, and the average score was found to be 56 and 57.21 for male and female controls, respectively. No average data was available in the Italian and Japanese studies. On the other hand, the study by Alimohammadi et al. [27] also reported a higher average score (74.93 ± 13.42 with 0–5 rating; it must have been 89.92 by 1–6 rating) for Persian nonindustrial employees. We may speculate that higher average scores found in Persian and Turkish studies could be related with the sociocultural differences in comparison to the Western countries; this aspect has never been questioned before and needs further research, which will be conducted in different countries. 

Beyond this difference, our data was in accordance with the previous reports revealing that NSS was not different between males and females, and within the age subgroups [2,3,6]. However, Senese et al. [28] reported that females and people older than 45 years were more noise sensitive than males and younger people, respectively.

van Kamp and Davies [11] pointed out importance of focusing of noise research on vulnerable groups which could be defined by socioeconomic and/or education status. Although noise exposure was found to be associated with income level [36], no direct data presenting a relationship between SNS and any measure of socioeconomic status was published. In our study, we used high school education as a cut–off and found that education after high school alone did not address any difference regarding increased or decreased risk of SNS, as reported by Moriera and Brian [2] and Belojevic and Jakovljevic [6]. Michaud et al. [37] reported that education level had statistically significant associations with traffic noise annoyance. 

As a conclusion, it is said that WNSS is convenient tool for evaluation of SNS in Turkey even if the average score was higher than reported in the Western countries. No difference was detected between males and females, age subgroups (< 55 years of age), and education level (with cut–off of high school).
